# Overexpression of *Acetyl CoA Carboxylase 1* and *3* (*ACCase1* and *ACCase3*), and *CYP81A21* were related to cyhalofop resistance in a barnyardgrass accession from Arkansas

**DOI:** 10.1080/15592324.2023.2172517

**Published:** 2023-02-01

**Authors:** Fidel González-Torralva, Jason K. Norsworthy

**Affiliations:** Department of Crop, Soil, and Environmental Sciences, University of Arkansas, Fayetteville, AR, USA

**Keywords:** Grass weed species, *Oryza sativa*, resistance to herbicides, rice, weed species

## Abstract

Barnyardgrass [*Echinochloa crus-galli* (L.) P. Beauv.] is the most difficult-to-control weed species of rice production systems worldwide. It has evolved resistance to different herbicide sites of action, including the acetyl-CoA carboxylase (ACCase)-inhibiting herbicides. Target-site mutations conferring resistance to ACCase-inhibiting herbicides are well documented; however, the role of the different *ACCase* genes in conferring resistance to cyhalofop-p-butyl (cyhalofop), an ACCase-inhibiting herbicide, remains poorly understood. This research assessed the contribution of gene amplification and expression of *ACCase* genes in a cyhalofop-resistant barnyardgrass accession. Additionally, the expression of *glutathione-S-transferases* (*GST*s) and cytochrome P450 monooxygenases (*P450*s) genes as possible contributors to resistance to cyhalofop were investigated. Results demonstrated that *ACCase* gene amplification does not contribute to cyhalofop resistance. However, *ACCase1* and *ACCase3* were found to be overexpressed in the cyhalofop-resistant barnyardgrass accession. At 24 h after cyhalofop treatment, an overexpression of 2.0- and 2.8-fold was detected in *ACCase1* and *ACCase3*, respectively. In addition, *CYP81A21* (a *P450* gene) was found to be 2.5-fold overexpressed compared to the susceptible accession in the same time period. These results suggest that *ACCase1, ACCase3*, and *CYP81A21* are crucial genes in contributing cyhalofop resistance in this barnyardgrass accession.

## Introduction

Barnyardgrass [*Echinochloa crus-galli* (L.) P. Beauv.] is a troublesome grass weed species in different cropping systems, but its presence is frequently related to rice (*Oryza sativa* L.) cultivation. In rice, the most prevalent weed species reported worldwide include barnyardgrass, weedy rice (*Oryza sativa* L.), and *Cyperus* weed species.^1^ Barnyardgrass is a hexaploid (2 n = 6x = 54) weed species.^2,3^ It possesses a remarkable ability to adapt to new environments and produces an exorbitant number of seeds (ranging from 34,600 to 39,000 seeds plant^−1^) that are easily dispersed by different means.^4,5^ In addition, barnyardgrass has allelopathic characteristics.^6^ Yield reductions in rice due to barnyardgrass vary depending on the conditions studied; however, yield reductions can be up to 70% [reviewed by ^6^].

Control of barnyardgrass relies on the use of herbicides, and unfortunately, many accessions with resistance to different molecules have been reported worldwide.^7^ In the US, herbicide-resistant barnyardgrass accessions exist in various states, including Arkansas, the largest rice-producing state in the US.^7–9^ Globally, barnyardgrass has evolved resistance to many herbicide sites of action, with some specific herbicide examples being propanil (photosystem II–inhibiting herbicide),^10^ pendimethalin (microtubule assembly-inhibiting herbicide),^7^ mefenacet (very long-chain fatty acid synthesis-inhibiting herbicide),^11^ quinclorac (auxin mimic-inhibiting herbicide),^12^ cyhalofop,^13^ penoxsulam (acetolactate synthase inhibiting-herbicide),^14^ glyphosate [5-enolpyruvylshikimate-3-phosphate synthase-inhibiting herbicide (*EPSPS*)],^15^ and clomazone (carotenoid biosynthesis-inhibiting herbicide).^16^

The acetyl CoA carboxylase (ACCase; EC. 6.4.1.2) is a crucial enzyme that catalyzes the carboxylation of acetyl-CoA to produce malonyl-CoA.^17^ In plants, ACCase is essential for the formation of primary fatty acids together with the buildup of long-chain fatty acids and flavonoids. ACCase-inhibiting herbicides work by blocking the formation of malonyl CoA, and, as a consequence, susceptible plants die from not having the ability to produce fatty acids.^17,18^ Necrosis of meristematic tissues, chlorosis and necrosis of apical meristem (flag leaf), and a purple leaf coloration due to the accumulation of anthocyanins are typical injury symptoms in plants treated with ACCase-inhibiting herbicides.^17,19^ Rice can safely tolerate cyhalofop due to a lack of esterase function, reduced absorption of the herbicide, and a rapid metabolism of cyhalofop acid.^20^

In barnyardgrass, a severe cessation of growth, chlorosis, and desiccation may be observed together with new leaf growth-inhibition.^21^ Nonetheless, resistant plants have deployed different strategies to avoid herbicide damage. Among those, plants have evolved a target-site mutation in the *ACCase* gene together with metabolic resistance, resulting in the herbicide being converted to nontoxic forms to surpass the herbicide toxicity.^17,18,22^ Metabolic resistance of herbicides is convoluted and is normally governed by superfamilies of GSTs and P450s. However, it also includes glucosyltransferases (GTs) and aldo-keto reductase enzymes. Those superfamilies have been correlated to herbicide metabolism in different plant species.^22–30^ In previous research, the metabolic resistance of barnyardgrass to cyhalofop herbicide was reported. The resistance in this population was attributed to a reduction in cyhalofop-acid compounds in the resistant accession compared to the susceptible standard.^13^

Here, the contribution of *ACCase* genes to cyhalofop resistance has been explored. Thus, the main objective of this research was to describe the target-site resistance mechanisms involved in a cyhalofop-resistant barnyardgrass accession collected in eastern Arkansas. Gene-specific *ACCase* primers were designed to study their role in gene expression, gene amplification, and assess their involvement in the observed resistance to cyhalofop herbicide. Additionally, the role of *GST*s and *P450*s genes as possible contributors to resistance to cyhalofop herbicide in barnyardgrass was examined.

## Materials and methods

### Plant material

A barnyardgrass accession previously characterized for resistance to cyhalofop and other aryloxyphenoxypropionate herbicides, together with a known susceptible accession were used in this research.^13^ Under greenhouse conditions, the resistant barnyardgrass accession displayed a resistance factor of ≈ 15 compared to the susceptible standard when treated with cyhalofop.^13^ To decrease biological variation among plants, the resistant accession followed an additional selection by treating plants with 3× cyhalofop field rate (1× = 313 g ai ha^−1^). Treatments were carried out using a lab track sprayer calibrated to deliver 187 L ha^−1^ using 1100067 nozzles. Further selected seeds were germinated using growing medium (Promix, LP15, Premier Horticulture Inc., PA, USA). When seedlings reached the one-leaf stage, they were transplanted to a 5 cm-diameter pot containing silt loam field soil (34% sand, 53% silt, 13% clay, and 1.5% organic matter). Plants were maintained under greenhouse conditions as described elsewhere.^13^

### ACCase-specific gene sequencing

Complementary DNA (cDNA) was extracted as described before.^31^ To describe the presence of target-site mutations, six specific primer sets, as described in ^32^, were used to partially amplify the putative six *ACCase* gene copies in the cyhalofop-resistant and -susceptible barnyardgrass accessions (Table 1). Primer sets covered the region where target-site mutations have been previously associated with resistance to ACCase-inhibiting herbicides. Polymerase Chain Reactions (PCRs) were performed using One*Taq* Hot Start 2× Master Mix with Standard Buffer (New England Biolabs, Ipswich, MA, USA) according to manufacturer directions and using 5 µL cDNA (five-fold dilution) as template in 25 µL reaction. To assess the best PCR conditions for *ACCase* amplification, gradient PCRs were carried out at different temperatures to select the optimum annealing temperature for each *ACCase* gene. Final PCR conditions were as follows: 94°C for 30s, 30 cycles of 94°C for 30s, 59–60°C for 30s, and 68°C for 135 s, a final extension of 68°C for 5 min, and reactions held at 10°C. After cycling, a 5 µL PCR product aliquot was loaded onto a 1.2% agarose gel to corroborate a single presence band. The remaining PCR products were cleaned using the Wizard SV Gel and PCR Clean up System (Promega Corp., Madison, WI, USA) and sent for bidirectionally Sanger sequencing (Eurofins Genomics, Louisville, KY, USA). Raw sequences were processed using BioEdit software.^33^ At least three biological replicates for each resistant and susceptible barnyardgrass accession were sequenced.

**Table 1.** Primer sets used to partially amplify the *ACCase* gene copies in cyhalofop-resistant and -susceptible barnyardgrass accessions.^32^

Gene^a^	Forward 5’ – 3’	Reverse 5’ – 3’	Annealing Temperature (°C)
*ACCase1*	TTAGGTGGATTATTGACTCTGTTGTG	TGGAATACAACAAAGGTACAACAACC	60
*ACCase2*	CAGCTGGATAGTGGGGAAAC	TGTTTTACCTCTGTCATGAAGTTACA	n/s
*ACCase3*	TTAGGTGGATTATTGACTCTGTTGTG	GAGCAAATTCAAATGCCGAAT	59
*ACCase4*	GCAGCTGGATAGTGGGGAAAT	GACAATCACGGATTTCAACAAC	n/s
*ACCase5*	CAGCTGGATAGTGGGGAAAC	GGAAAGGCTAGTTTTACCTCTTTG	59
*ACCase6*	GCAGCTGGATAGTGGTGAAG	GTGATTCATGAACAGTACCATCTGC	n/s

^a^*ACCase, acetyl CoA carboxylase*; n/s, not estimated.

### Gene expression under metabolic inhibition

Plants of resistant and susceptible accessions were managed as described in “Plant material” section. Plants were treated at the three- to four-leaf growth stage with either malathion (a P450 inhibitor) or NBD-Cl (a GST inhibitor) to assess *ACCase, GST*s, and *P450*s gene expression under metabolic inhibition. Treatments consisted of a non-treated control (T1); cyhalofop at 313 g ai ha^−1^ (T2); malathion at 2 kg ai ha^−1^ + cyhalofop at 313 g ai ha^−1^ (T3); and NBD-Cl at 80 g ha^−1^ followed by cyhalofop at 313 g ai ha^−1^ (T4) 48 h later. NBD-Cl was dissolved on 1% acetone + 0.1% Tween 20.^24^ Cyhalofop treatments contained 1% v/v crop oil concentrate (Agri-dex, Helena Chemical Company, Collierville, TN, USA). Each pot had two seedlings which was considered one replicate, and each treatment had three replicates (*n* = 6).

### RNA extraction and complementary DNA (cDNA) synthesis

RNA extraction and complementary DNA (cDNA) synthesis were performed as described before.^31^ Fresh tissue of resistant and susceptible barnyardgrass accessions was collected, placed in 2 mL Eppendorf tubes, and immediately frozen in liquid nitrogen. Samples were stored at −80°C until RNA extraction. Monarch Total RNA Miniprep kit (New England Biolabs, Ipswich, MA, USA) was used to extract the total RNA. RNA quality and quantity were assessed spectrophotometrically using a nanodrop (Nanodrop 2000c, Thermo Scientific, Waltham, MA, USA). cDNA was obtained with 1 µg of total RNA as template using the iScript Reverse Transcription Supermix kit (Bio-Rad Laboratories Inc., Hercules, CA, USA).

### ACCase gene expression

Transcripts were measured in non-treated and treated plants of resistant and susceptible barnyardgrass accessions. Thus, tissue of non-treated and treated plants was collected 24 h after treatments (HAT) for total RNA and cDNA synthesis as described earlier. Quantitative PCR (qPCR) was utilized to measure the transcript levels using cDNA according to MIQE guidelines.^34^ Homologs of *β-actin* and *glyceraldehyde 3-phosphate dehydrogenase* (*GAPDH*) genes in barnyardgrass were used as reference genes to estimate the basal transcript levels in both treated and non-treated plants of resistant and susceptible accessions^28,35,36^ (Table 2). Previously research in barnyardgrass (*E. crus-galli* var. *formosensis*) resistant to cyhalofop reported the presence of six *ACCase* gene copies.^32^ These six copies of *ACCase* genes were used in this study as target genes. Nucleotide sequences of *ACCase* gene copies deposited at the National Center for Biotechnology Information (NCBI) were retrieved to design a set of gene-specific primers. Additionally, a set of primers common to all six *ACCase* gene copies was designed based on a highly conserved region. Primers were designed using the Primer3Plus software^37^ (Table 2). A CFX Connect Real-Time System instrument provided with CFX Maestro software (Bio-Rad Laboratories Inc., Hercules, CA, USA) was used to run all qPCR reactions and retrieve the quantification cycle (Cq) values, respectively. Each qPCR reaction comprised 5 µL of SsoAdvanced Universal SYBR Green Supermix (2×) (Bio-Rad Laboratories Inc., Hercules, CA, USA), 0.3 µL of each forward and reverse primer at 10 µM, 2.9 µL deionized water, and 1.5 µL of a 5-fold dilution cDNA in a 10 µL total volume ran on 96-well plates. The amplification protocol consisted of 30 sec at 98°C followed by 40 cycles of 10 sec at 98°C and 30 sec at 61°C and finalized with a standard melt curve analysis to corroborate correct amplification. No-template controls (deionized water instead of cDNA) on each primer set were included in every qPCR run. Amplicon efficiency for each primer set and sample reactions was obtained using the LinRegPCR software version 2021.2.^38^ Fold-change expression values were calculated using the formula:

$$Relative\;gene\;expression=\frac{{\left(E_{TG}\right)}^{\Delta CqTG}}{GeoMean[\left(E_{RG}{)}^{\Delta CqRG}\right]}$$

**Table 2.** Primer sequences of genes used to measure either transcript abundance or gene copy number in resistant and susceptible barnyardgrass accessions by qPCR.

Gene ^a^	Sequence 5’ → 3’ ^b^	Amplicon (bp)	Slope	Efficiency (%) ^c^
*ACCase1-6*^d^	F CATGGAAGTGCTGCTATTGCC R TACCAAGCCGAGCAAGATAAGC	115	−3.175	106.5
*ACCase1*	F GTAGTTCTGTGATCTAGCCAGCG R ACCAAAGGTACAACAACCAACAG	193	−3.150	107.7
*ACCase2*	F GCCTTTCCATGATTGTCATGTGG R GCAATGACCTACAGCAGGACC	111	−3.292	101.3
*ACCase3*	F TCTATGATCTAGCCAGCGGTTC R TCCGAGCAAATTCAAATGCCG	176	−3.417	96.2
*ACCase4*	F TTGTTGAATTAGCATTCGGCATTAG R AGAGATGTCTGAATATGTTCCCAG	138	−3.182	106.2
*ACCase5*	F GTATTTACCTGCGGTTCCATTCTG R ACCTCTTTGTCATGAAGCTAAGG	194	−3.379	97.7
*ACCase6*	F GGTGCAGTCAGAGGAAGGTG R ACTGCACGAGATCTGAACCG	153	−3.207	105.1
*CYP81A21*	F GACGGTGAGAAGAAGAGCATG R AACACTCACCGCACATAGTG	105	−3.364	98.3
*CYP81A68*	F CTCCCGCCTGGTCACCG R GCAGTCGGCGGAGGACA	124	−3.128	108.8
*GST1*	F TCCTGCCTGTTCTGGTTTGAG R CTCCGCACTTTTCCCAAATCG	114	−3.210	104.9
*GST1a*	F ATATCCTGCGCATCCTCTTCTG R CCCTCATGCAAACCAATAACGG	126	−3.312	100.4
*GST2*	F GTCAGGTTCCAGCTTTGCAAG R GATTGCCTTCCTTCAACAGCTC	108	−3.253	102.9
*GST2c*	F TGTACGAGTGCCTCATCAACC R TCGTAGACATCGAGCACCTTC	103	−3.156	107.4
*GSTL3*	F AGGCGTCGATCTTGTTCATCTC R GCGGATATTGCATACGTGACG	130	−3.343	99.1
*β-actin*	F CTGTTCCAGCCATCGTTCATTG R ATACCTGGGAACATCGTGGAAC	146	−3.304	100.8
*GAPDH*	F AACGCTCTACTGGGTCTTGAAG R GACAACTTCGTCAAGCTCGTC	102	−3.257	102.8
*CCR*	F GTGCTCACGTCGTCCATCG R GCTCTTGCAGAAGTCGAGGTC	102	−3.324	99.9
*PPAN*	F CAGATCACGGGTGCCAAGAT R AGTGTAGGGGAGCATGACCT	111	−3.203	105.2

^a^*ACCase, acetyl CoA carboxylase; CYP, cytochrome P450 monooxygenase; GST, glutathione-S-transferase; GSTL, glutathione S-transferase lambda; GAPDH, glyceraldehyde 3-phosphate dehydrogenase; CCR, cinnamoyl-CoA reductase; PPAN, peter Pan-like*. ^b^ F, forward primer; R, reverse primer. ^c^ Primer efficiency in gene copy number experiments was determined using E = [10^(−1/slope)^ – 1] × 100; for gene expression experiments efficiency was obtained using LinRegPCR software. ^d^ Common primer to *ACCase1-ACCase6*.

where *E* corresponds to base of exponential amplification, ΔCq *TG* is the difference in the quantification cycles (*Cq*) of the target gene (*TG*), GeoMean represents the geometric mean of both reference genes (*RG*) (*β-actin* and *GAPDH*), and ΔCq *RG* is the difference in quantification cycles of reference genes.^39^

To reduce biological variation among samples, equal amounts of leaf tissue from two plants were pooled to generate a single biological replicate. Subsequently, three biological replicates (*n* = 6) were obtained and further processed. A Student’s *t*-test was used to detect significant differences in fold-change expression between resistant and susceptible barnyardgrass accessions on each treatment.

### P450s and GSTs gene expression

Homologs of the *CYP81A21, CYP81A68, GST1, GST1a, GST2, GST2c*, and *GSTL3* genes in barnyardgrass were retrieved from the NCBI database. Primer design followed the same methodology as described earlier. Such genes have been reported in conferring resistance to different ACCase-inhibiting herbicides in Asia minor bluegrass (*Polypogon fugax* Nees ex Steud.), barnyardgrass, late watergrass [*Echinochloa phyllopogon* (Stapf) Koso-Pol.], and rigid ryegrass (*Lolium rigidum* Gaudin).^23,26–28^

### ACCase gene copy number

Genomic DNA (gDNA) extracted from resistant and susceptible barnyardgrass was used to estimate the gene copy number of the *ACCase* genes relative to two reference genes using a qPCR approach and adhering to MIQE guidelines^34^ as described in the “*ACCase*-specific gene sequencing” section. Homologs of the *cinnamoyl-CoA reductase* (*CCR*) and *peter Pan-like* (*PPAN*) genes in barnyardgrass were used as reference genes (Table 2). Such genes have been reported as a single gene copy in different plant genomes.^40,41^ Gene copy number estimation was carried out as described before.^31^ The gDNA was extracted from resistant and susceptible barnyardgrass accessions using the E.Z.N.A. Plant DNA kit (Omega Bio-Tek Inc., Norcross, GA, USA) and diluted to 10 ng µL^−1^ to use as template in subsequent qPCR reactions. Standard curves were created using 5-fold gDNA dilutions, and the efficiency (E) for each primer set was calculated as E = [10^(−1/slope)^-1] × 100.^42^ Each qPCR reaction included the same reagents as described earlier but 15 ng gDNA and run under the same conditions. The amplification protocol included 3 min at 98°C followed by 40 cycles of 10 sec at 98°C and 30 sec at 61°C and finalized with a standard melt curve analysis to corroborate correct amplification. Relative *ACCase* gene copy number was expressed as 2^ΔCq^ as described elsewhere.^43,44^ The experiment included four plants (*n* = 4) per accession, and each run included two technical replicates for each gene. A Student *t*-test was used to identify differences between resistant and susceptible barnyardgrass accessions.

## Results and discussion

### ACCase-specific gene sequencing

**Figure 1.**
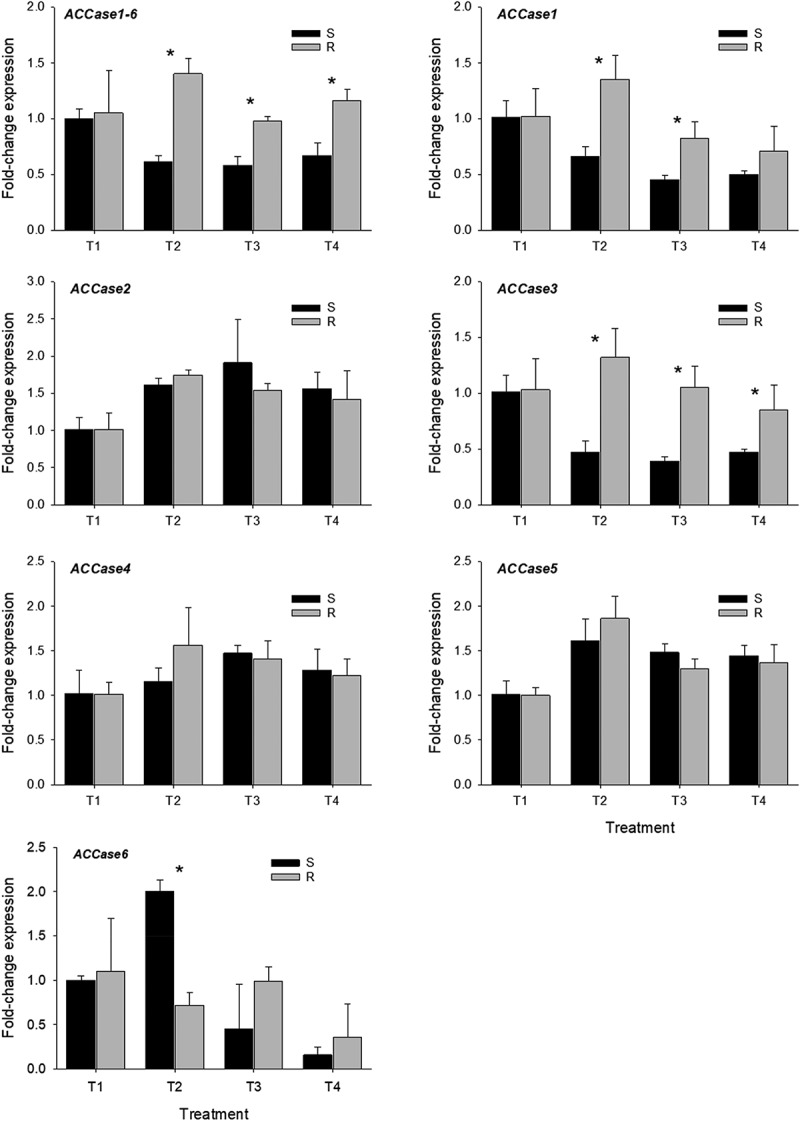
Quantitative PCR analysis of *acetyl CoA carboxylase* (*ACCase*) genes in susceptible (S) and resistant (R) barnyardgrass accessions under different treatments. Bars ± standard deviation of the mean. A significant difference between susceptible and resistant barnyardgrass accession on each treatment is indicated by an asterisk (*P* ≤ .05).

Target-site mutations in the *ACCase* gene have been previously reported as responsible for conferring resistance to ACCase-inhibiting herbicides.^17^ Those target-site mutations can be found commonly at positions 1781, 1999, 2027, 2041, 2078, 2088, and 2096 (numbered respect to that of *Alopecurus myosuroides*: GenBank accession AJ310767.1), all in the carboxyl transferase domain of ACCase enzyme.^18^ This research attempted to sequence all six *ACCase* gene copies previously described in barnyardgrass.^32^ Under the lab conditions, primers to amplify *ACCase2, ACCase4*, and *ACCase6* did not yield any product amplification compared to *ACCase1, ACCase3*, and *ACCase5* while using annealing temperatures ranging from 53 to 59°C (Figure 1 Supplementary) in the barnyardgrass accessions investigated. Consequently, those were discarded for furthersequencing investigation. The lack of product amplification using the same primers could be related to genetic diversity. *Echinochloa crus-galli* var. *crus-galli* is native to Central America, East Asia, and Europe, whereas *E. crus-galli* var. *formosensis* is native to East Asia.^45^ It is important to note that the number of *ACCase* genes in barnyardgrass remains unclear since some researchers have described different numbers of copies in the *ACCase* gene of this plant species. For instance, ref. ^35^ sequenced four copies of the *ACCase* gene from barnyardgrass.

In this research, analysis of the *ACCase1, ACCase3*, and *ACCase5* between resistant and susceptible accessions did not show any nucleotide change that could lead to a target-site mutation (no further details). Similar results have been published in other barnyardgrass accessions and other ACCase-resistant weed species. A target-site mutation was shown as an unlikely resistance mechanism in cyhalofop-resistant barnyardgrass.^13^ In another barnyardgrass population resistant to cyhalofop, no target-site mutation was reported,^32^ and similar findings were reported by ^46^, where a target-site mutation was rejected as a possible resistance mechanism in a multiple herbicide-resistant barnyardgrass accession from Ningxia province in China. Asia minor bluegrass resistant to quizalofop-p-ethyl (quizalofop) had no target-site mutation in any of the four *ACCase* genes reported.^23^ In large crabgrass [*Digitaria sanguinalis* (L.) Scop.] resistant to clethodim, sethoxydim, fenoxaprop-p-ethyl (fenoxaprop), fluazifop-p-butyl, and quizalofop herbicides, no target-site mutation was found in the *ACCase* gene.^47^

### Gene expression under metabolic inhibition

In this study, resistant and susceptible barnyardgrass plants were treated with malathion and NBD-Cl to assess *ACCase, GST*s, and *P450*s gene expression under metabolic inhibition. Treatments included a non-treated control (T1), cyhalofop at 313 g ai ha^−1^ (T2); malathion at 2 kg ai ha^−1^ + cyhalofop at 313 g ai ha^−1^ (T3); and NBD-Cl at 80 g ha^−1^ followed by cyhalofop at 313 g ai ha^−1^ 48 h later (T4). *ACCase* gene-specific primers were designed to describe their role in the observed resistance of barnyardgrass to cyhalofop. Additionally, genes from the literature reported to contribute to herbicide resistance in other weed species were chosen. At 24 HAT, which was the time set up for sample collection, there were differences between resistant and susceptible accessions (*P* ≤ .05) (Figure 1). The rationale in having selected 24 HAT for qPCR experiments is based on the production of cyhalofop-acid, thus differences were found at this time period between resistant and susceptible accessions. Resistant accession produced lower amounts of cyhalofop-acid compared to the susceptible accession and no differences were detected after 24 h time period.^13^

Gene expression of *ACCase1-6* differed between resistant and susceptible accessions in treatments T2, T3, and T4 (*P* ≤ .05). Fold-change expression in the resistant accession was approximately 2-times higher than the gene expression found in the susceptible one (Figure 1, *ACCase1-6*). Ref. ^35^, reported similar results to those described here while using a common ACCase primer. At 6 h after cyhalofop treatment, there were differences in the expression level of resistant and susceptible accessions. Resistant accession was found to be 3.3-times overexpressed compared to susceptible, and the same trend was also observed in fenoxaprop- and pinoxaden-treated plants during the same time period.^35^ However, the specific *ACCase* gene causing such overexpression remains unspecified.

In a quizalofop-resistant barnyardgrass accession treated with quizalofop at 60 g ai ha^−1^, the basal gene expression in the resistant accession remained similar (≈ 1-fold) to that found in the non-treated control during the time of the study (8 days). However, in the susceptible accession, the gene expression decreased after herbicide application, and by 8 d after treatment, it was practically null.^48^ In that research, it was concluded that gene overexpression was not involved in the resistance mechanism, but the number of *ACCase* genes present in such accessions was unclear.^48^ Gene overexpression has been reported as well in johnsongrass [*Sorghum halepense* (L.) Pers.] resistant to sethoxydim and quizalofop, whereby the resistant accession displayed approximately 2.5-fold higher ACCase enzyme activity compared to susceptible accession.^49^

*ACCase1* also displayed differences in T2 and T3 treatments (*P* ≤ .05), where the resistant accession had approximately 2-fold more expression than the susceptible used for comparison (Figure 1, *ACCase1*). Similarly, *ACCase3* in the resistant barnyardgrass showed 2.8-, 2.7-, and 1.8-fold more expression (*P* ≤ .05) than the susceptible for T2, T3, and T4, respectively (Figure 1, *ACCase3*). Expression levels in *ACCase2, ACCase4*, and *ACCase5* did not differ among treatments (Figure 1, *ACCase2, ACCase4*, and *ACCase5*). Surprisingly, in *ACCase6*, susceptible barnyardgrass had a 2.8-fold increase in expression than the resistant accession (*P* ≤ .05) in T2 treatment. Such overexpression would result from ACCase inhibition and stress caused by cyhalofop herbicide in this susceptible accession.^50^ Nonetheless, it is interesting to note that the expression levels in both susceptible and resistant accessions in T3 and T4 treatments remained mostly below that of non-treated controls (T1) (Figure 1, *ACCase6*). Interestingly, expression levels observed in the susceptible accession in *ACCase1, ACCase3*, and *ACCase6* mainly in T3 and T4 treatments were lower than those found in the resistant accession. That could be explained by the presence of the metabolic inhibitors in those treatments that “enhanced” the efficacy of cyhalofop. Additionally, results suggest the involvement of metabolic resistance in the cyhalofop-resistant barnyardgrass. Expression levels found using cyhalofop alone *versus* in combination with the metabolic inhibitors decreased mainly in *ACCase1* and *ACCase3*. Perhaps follow up gene expression experiments should explore longer harvest time points to gain more details about *ACCase* gene profiles. On the other hand, the detection of transcripts in *ACCase2, ACCase4*, and *ACCase6* can be explained by the high sensitivity and specificity by qPCR technique along with the detection limits compared to a conventional PCR.^51,52^

**Figure 2.**
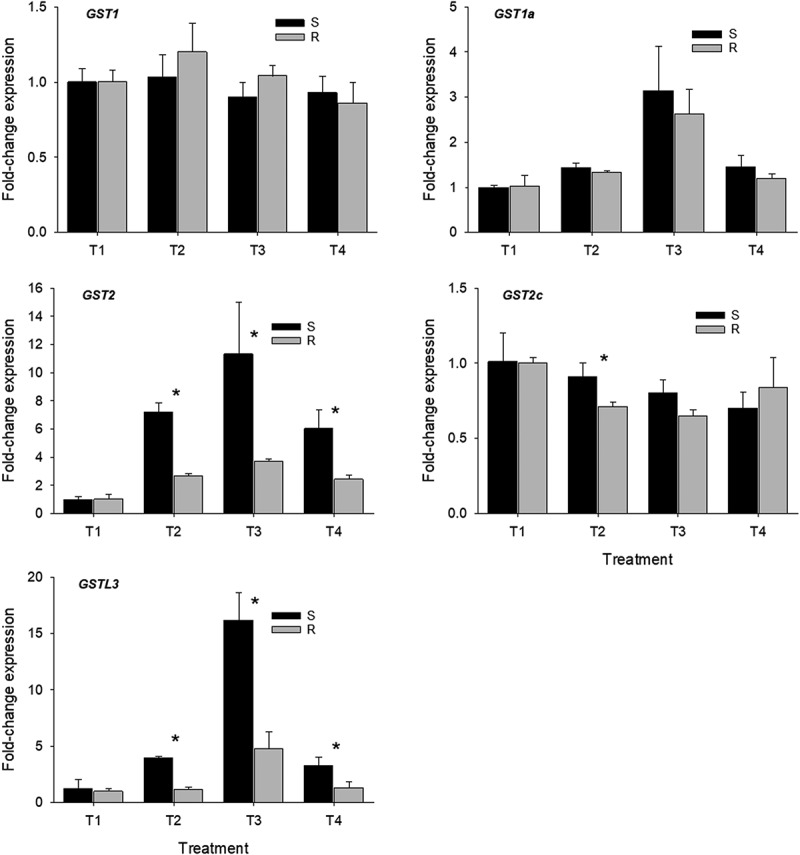
Quantitative PCR analysis of glutathione-*S*-transferase (*GST*s) genes in susceptible (S) and resistant (R) barnyardgrass accessions under different treatments. Bars ± standard deviation of the mean. A significant difference between susceptible and resistant barnyardgrass accession on each treatment is indicated by an asterisk (*P* ≤ .05).

Expression levels using the *GST1* and *GST1a* had no differences between accessions in the treatments evaluated (Figure 2, *GST1, GST1a*). Interestingly, *GST2* was overexpressed by 2.7-, 3.0-, and 2.5-fold (*P* ≤ .05) in the susceptible accession in T2, T3, and T4 treatments, respectively (Figure 2, *GST2*). A similar response was obtained with *GST2c* and *GSTL3* genes. In *GST2c*, treatment T2 caused a higher expression (1.3-fold) level in the susceptible accession compared to that of the resistant (*P* ≤ .05) (Figure 2, *GST2c*). In *GSTL3*, susceptible accession displayed 3.4-, 3.3-, and 2.5-fold higher expression levels in T2, T3, and T4 treatments, respectively compared to that found in the resistant accession (*P* ≤ .05) (Figure 2, *GSTL3*). Contrary to the results found in this research, in a quizalofop-resistant accession of Asia minor bluegrass with no herbicide treatment, authors reported that *GST2c* and *GSTL3* were constitutively expressed in the resistant accession. The authors concluded that quizalofop resistance in Asia minor bluegrass is related to non-target-site resistance mechanisms and that *GST2c* and *GSTL3* are involved in resistance.^23^

**Figure 3.**
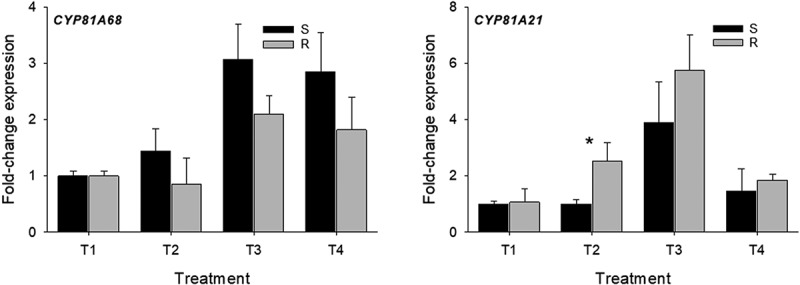
Quantitative PCR analysis of *CYP* P450 genes in susceptible (S) and resistant (R) barnyardgrass accessions under different treatments. Bars ± standard deviation of the mean. A significant difference between susceptible and resistant barnyardgrass accession on each treatment is indicated by an asterisk (*P* ≤ .05).

Regarding the *P450*s genes analyzed, gene expression using the *CYP81A68* did not differ between susceptible and resistant accessions in the treatments evaluated (Figure 3, *CYP81A68*). Nonetheless, in other barnyardgrass accessions resistant to ALS and ACCase-inhibiting-herbicides, it has been reported that the overexpression of *CYP81A68* confers metabolic resistance to penoxsulam (an ALS-inhibiting herbicide) but also can confer resistance to cyhalofop and metamifop (ACCase-inhibiting herbicides).^28^ Using the *CYP81A21*, the gene expression in the resistant accession showed a 2.5-fold increase in T2 treatment compared to that found in the susceptible accession (*P* ≤ .05) (Figure 3, *CYP81A21*). This result suggests that *CYP81A21*, a *P450* gene previously reported in contributing to resistance in late watergrass, is also contributing to resistance in those barnyardgrass accessions. Overexpression of *CYP81A21* gene has resulted in an increase in cross-resistance to ALS and ACCase-inhibiting herbicides.^27^ The accessions used in this research were both susceptible to the ALS-inhibiting herbicide imazethapyr-ammonium.^13^ These results suggest that genes involved in metabolic resistance in one plant species not precisely should be involved in another one. For instance, *GST2c* and *GSTL3* were discarded in this research as contributing to ACCase resistance. However, in Asia minor bluegrass, both genes were involved in the resistance to quizalofop.^23^ Additionally, *CYP81A68* was not associated to cyhalofop resistance in this research but it was in other barnyardgrass accessions.^28^ Conversely, *CYP81A21* was linked to cyhalofop resistance in this study but also in late watergrass.^27^

Metabolism of ACCase-inhibiting herbicides has been reported as the non-target-site resistance mechanism in several resistant weed species, including Asia minor bluegrass resistant to quizalofop,^23^ Chinese sprangletop [*Leptochloa chinensis* (L.) Nees] resistant to cyhalofop,^30^ barnyardgrass resistant to penoxsulam, cyhalofop and metamifop,^25,28^ and rigid ryegrass resistant to multiple herbicide sites of action.^26^ However, in ALS-resistant accessions of barnyardgrass and late watergrass, both target and non-target-site resistance mechanisms were reported. Resistance was attributed to an Ala122Asn and Trp574Leu target-site mutations in the *ALS1* gene and overexpression of the *ALS1* and no *ALS2* or *ALS3* copies.^53^

Gene overexpression has been correlated to herbicide resistance in different herbicide-resistant weed species, which can result in an increased amount of enzyme produced by the plant to avoid herbicide damage. Thus, gene overexpression can be due to regulatory changes that increase gene transcription and/or a difference in the genomic copy number of the target gene, which also causes an increased gene transcription.^22^ Results obtained in this research support the idea that gene overexpression of specific *ACCase* genes contributes to cyhalofop resistance in those barnyardgrass accessions. Rapid detection of herbicide-resistant weed species is crucial to implement the best possible weed management strategies.

### ACCase gene copy number

**Figure 4.**
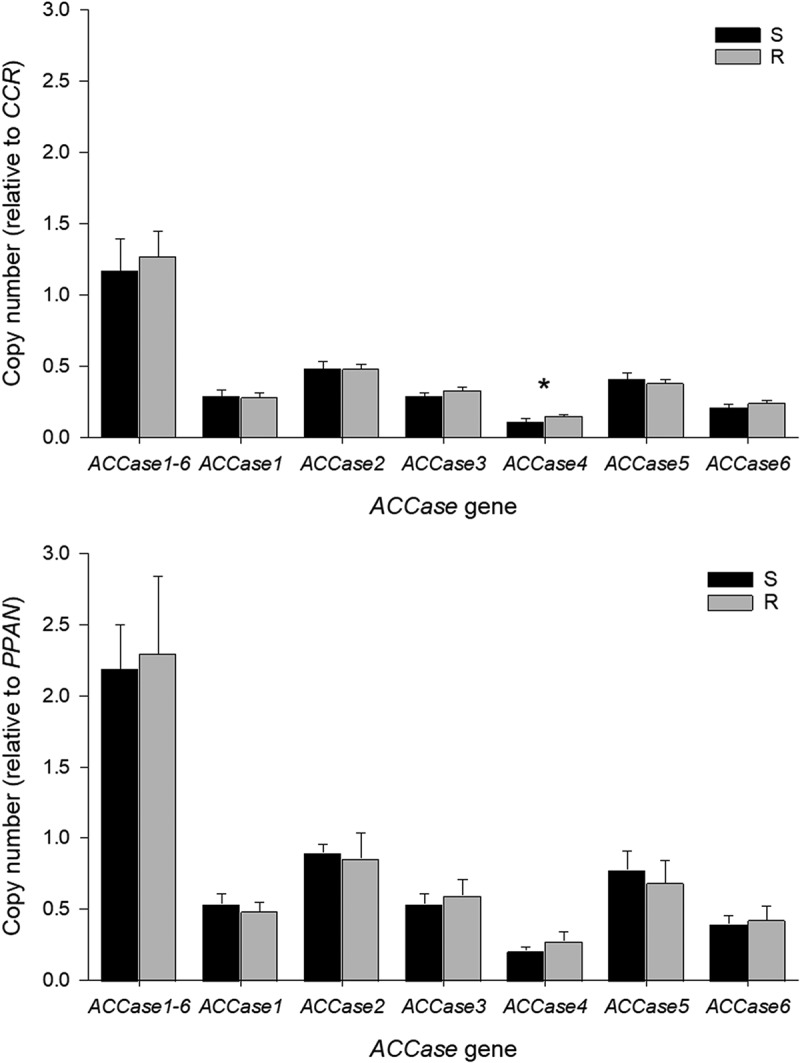
Relative gene copy number estimation of *acetyl CoA carboxylase* (*ACCase*) genes in susceptible (S) and resistant (R) barnyardgrass accessions. *CCR* and *PPAN* were used as reference genes to measure the relative gene copy number. Bars ± standard deviation of the mean. A significant difference between susceptible and resistant barnyardgrass accession on each gene is indicated by an asterisk (*P* ≤ .05).

Results displayed a low number of copies in all gene-specific *ACCase* compared to *CCR* and *PPAN* genes. Relative to *CCR*, the resistant accession showed a higher copy number (*P* ≤ .05) in the *ACCase4* gene. However, biologically, this difference could not be related to ACCase resistance. Using *PPAN*, the relative gene copy number in susceptible and resistant accessions among *ACCase* genes, had no differences (*P* ≥ .05) (Figure 4).

Gene amplification of the target-site gene has conferred herbicide resistance in a diverse range of herbicide-resistant weed accessions.^22^ Gene amplification of the *EPSPS* gene (target-site of glyphosate herbicide) in a diverse range of glyphosate-resistant weed species has been reported as a common target-site resistant mechanism.^22^ Other than glyphosate, gene amplification has been found in more herbicide-resistant accessions with different herbicide sites of action. For instance, in Palmer amaranth (*Amaranthus palmeri* S. Watson) resistant to glufosinate, the resistant mechanism deployed was attributed to the amplification of the chloroplastic *glutamine synthetase* (*GS*) gene.^54^ In large crabgrass accessions resistant to ACCase-inhibiting herbicides, gene amplification of the target *ACCase* gene was also reported.^47^ In both cases, overexpression of the target-site gene was due to gene amplification.

In summary, this research focused on deciphering the role of gene expression and gene amplification of specific *ACCase* genes on cyhalofop resistance in barnyardgrass. Previously, the presence of a non-target-site resistance mechanism was documented, where a difference in cyhalofop-acid formation was detected between resistant and susceptible accessions at 24 h after cyhalofop treatment.^13^ In this research, it has been demonstrated that *ACCase1* and *ACCase3* genes contribute to cyhalofop resistance. Furthermore, the overexpression observed in *CYP81A21* suggests the involvement of this gene in cyhalofop resistance. Additionally, results indicate that gene amplification of *ACCase* genes is not contributing to resistance in this cyhalofop-resistant barnyardgrass accession.

## Supplementary Material

Supplemental Material
