# Supplementary figures and images for: Overexpression of Acetyl CoA Carboxylase 1 and 3 (ACCase1 and ACCase3), and CYP81A21 were related to cyhalofop resistance in a barnyardgrass accession from Arkansas
Source: Plant Signal Behav. 2023 Feb 1;18(1):2172517. doi: 10.1080/15592324.2023.2172517 (PMC9897766; doi:10.1080/15592324.2023.2172517)

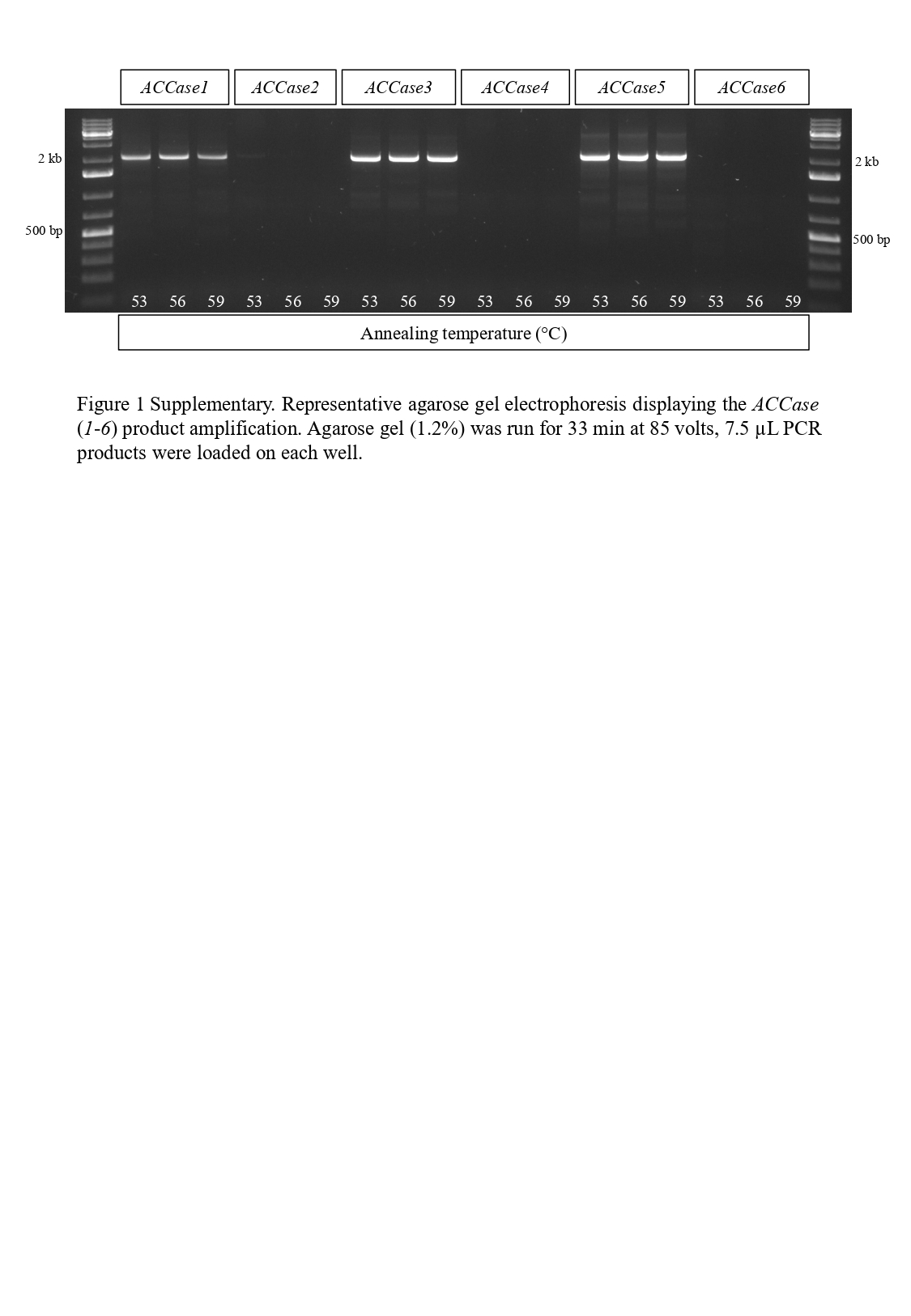

Supplement: Supplemental Material [file KPSB_A_2172517_SM2789.jpg]
